# Risk of Abdominal Obesity Associated with Phthalate Exposure of Nurses

**DOI:** 10.3390/toxics10030143

**Published:** 2022-03-18

**Authors:** Branislav Kolena, Henrieta Hlisníková, Ľubica Kečkéšová, Miroslava Šidlovská, Tomáš Trnovec, Ida Petrovičová

**Affiliations:** 1 Department of Zoology and Anthropology, Constantine the Philosopher University in Nitra, 94974 Nitra, Slovakia; hhlisnikova@ukf.sk (H.H.); lubica.keckesova@student.ukf.sk (Ľ.K.); msidlovska@ukf.sk (M.Š.); ipetrovicova@ukf.sk (I.P.); 2 Department of Environmental Medicine, Slovak Medical University, 83303 Bratislava, Slovakia; tomas.trnovec@szu.sk

**Keywords:** nurses, occupational exposure, phthalates, obesogenic effect, health risk

## Abstract

Background: Occupational health hazards associated with phthalate exposure among nurses are still not well understood. Methods: We used high-performance liquid chromatography and tandem mass spectrometry to analyze phthalates. Anthropometric measurements and questionnaires were conducted. Results: We observed associations between mono-benzyl phthalate (MBzP) and body mass index (BMI), hip circumference (HC), waist circumference (WC), waist to height ratio (WHtR), and fat mass index (FMI), visceral fat content, BMI risk and hip index risk (HIrisk), adjusted to consumer behavior and consumer practices (r = 0.36–0.61; *p* ≤ 0.046). In the same model, we detected an association between mono-n-butyl phthalate (MnBP) and waist to hip ratio (WHR; r = 0.36; *p* = 0.046), mono-carboxy-isononyl phthalate (cx-MiNP) and BMI (r = 0.37; *p* = 0.043), HC (r = 0.4; *p* = 0.026) and WHtR (r = 0.38; *p* = 0.037), between mono-oxo-isononyl phthalate oxo (MiNP) and HC (r = 0.36; *p* = 0.045), mono-2-ethylhexyl phthalate (MEHP), mono(2-ethyl-5-oxohexyl) phthalate (oxo-MEHP) and HIrisk (r = 0.38–0.41; *p* ≤ 0.036), between oxo-MEHP and Anthropometric Risk Index (ARI risk; r = 0.4; *p* = 0.028). We detected a relationship between BMI and MBzP (β = 0.655; *p* < 0.001) and mono-2-ethylhexyl phthalate (MEHP; β = −0.365; *p* = 0.003), between hip circumference and MBzP (β = 0.486; *p* < 0.001), MEHP (β = −0.402; *p* = 0.001), and sum of secondary metabolites of diisononyl phthalate (∑DiNP; β = 0.307; *p* = 0.016). We observed a relationship between fat content and MBzP (β = 0.302; *p* = 0.033), OH-MnBP (β = −0.736; *p* = 0.006) and MiBP (β = 0.547; *p* = 0.046), visceral fat content and MBzP (β = 0.307; *p* = 0.030), HI-risk and MBzP (β = 0.444; *p* = 0.001), ARI-risk and sum of di-n-butyl phthalate metabolites (∑DnBP; β = 0.337; *p* = 0.018). We observed an association between the use of protective equipment with cx-MiNP. Conclusions: Occupational exposure to phthalates may induce abdominal obesity and result in obesity-related metabolic disorders.

## 1. Introduction

The actual COVID era points to a long-overlooked status among front-line healthcare workers, especially nurses, in the context of the elimination of health-related risks. Nurses are an indispensable part of the health system in all countries committed to caring for patients. However, at the same time, nurses seem to be at increased occupational risk in several significant indicators such as for obesity, higher levels of stress, and lack of sleep.

The increasing prevalence of obesity is a major health concern [1,2,3] which does not pass over nurses [4,5]. Eating behavior, sleep deprivation, psychological, genetic, but also environmental, and behavioral stimuli play a part in obesity cofactors [6,7].

Anthropometrics is crucial in defining and assessing of abdominal obesity, and new anthropometric indices such as ABSI, HI, or ARI bring new insight into diagnosis and treatment options [8]. Body roundness index (BRI) allows for determining the human figure shape as an ellipse, generated from the height and waist circumference. BRI values range from 1 to 16, and individuals with a more rounded figure are characterized by greater BRI values. BRI is a predictor of the percentage of adipose tissue and visceral tissue and can be a useful tool in the assessment of health status [9]. The Clinica Universidad de Navarra-Body Adiposity Estimator (CUN-BAE) index was suggested to evaluate the percentage of fat content in the body and is calculated using BMI, sex, and age. Fat percentage calculated using CUN-BAE showed a strong correlation with the real content of adipose tissue [10].

Evidence suggests that interactions between environmental and genetic factors can lead to acquired obesity [11,12]. Phthalates belong to endocrine disrupting chemicals (EDCs) [13] and, as toxic environmental factors, have been known to follow non-monotonic dose-response curves [14,15]. Phthalates and bisphenol A (BPA), such as potential obesogens, are substances of particular concern commonly found in medical devices [16,17]. Sources of phthalates are medical utilities produced from polyvinylchloride (PVC), e.g., infusion and transfusion sets, sets for hemodialysis, parenteral nutrition, and oxygen masks [18].

The purpose of this study was to identify the potential links between occupational phthalate exposure and overweight/obesity among nurses who worked in psychiatric and mental health wards in hospital.

## 2. Materials and Methods

The study consisted of native Slovak adult subjects-nurses who worked in psychiatric and mental health wards in hospital (females, *n* = 50) recruited in September 2020 as a pooled cohort, consisting of volunteers from departments of the Philippe Pinel Psychiatric Hospital in Pezinok (Slovakia). 

The study was approved by the Institutional Review Board of the Philippe Pinel Psychiatric Hospital in Pezinok, Slovakia. Physical examination, questionnaires, and post-shift urine samples were collected. Participants signed informed consent before the study, and it was possible to withdraw participation anytime during the study. Subjects diagnosed with an illness or metabolic disorders (medications might be a source of high exposure to some phthalates), and with an incomplete questionnaire were excluded.

### 2.1. Anthropometry 

All anthropometric measures were performed by a trained researcher using a standardized protocol. During the anthropometric measurements, participants were light clothing and barefoot. Height (the vertical distance from the plane where the subject stands barefooted to the vertex on the head with their back), weight, waist circumference (measured in the horizontal plane at the mid-point between the anterior iliac crest and the inferior margin of the rib, using a tape measure), and hip circumference (measured at the level of the widest circumference over the great trochanters) were measured to the nearest 0.1 cm, 0.1 kg, and 0.5 cm, respectively. BMI was calculated as weight (kg)/height (m)^2^. Body composition (weight, body fat percentage, muscle mass percentage, and visceral fat level) was estimated by The Omron BF510 (Kyoto, Japan). Visceral fat level was estimated by Omron and describe intra-abdominal fat present around abdominal viscera in mesentery and omentum. Fat content means conversion of body fat percentage to kilograms.

WHtR was calculated as the waist circumference (cm) divided by the height (cm), waist to hip ratio (WHR) was calculated as dividing waist circumference (cm) by hip circumference (cm), FMI, and FFMI were calculated by standard anthropological procedure [19,20].

FMI was calculated as FM in kilograms divided by stature in square meters (kg/m^2^) and FFMI was fat free mass in kilograms divided by stature in square meters (kg/m^2^).

ABSI was estimated by formula: ABSI = WC/(BMI (2/3) × height (1/2)) [8]. HI and ARI were calculated according to Krakauer & Krakauer [21]. ABSI calculator is free available at https://nirkrakauer.net/sw/absi-calculator.html (accessed on 16/11/2021), and ARI calculator at https://nirkrakauer.net/sw/ari-calculator.html (accessed on 10/01/2021).

In BMI, ARI, ABSI and HI, the “z score” is define as: (value-mean)/standard deviation, where the mean and standard deviation are age and sex specific.

In BMI, ARi, ABSi and HI, “risk” define the death rate associated with given indices, normalized (risk of 1 = average death rate; risk of 2 = double the average death rate, etc.). A relative risk greater than 1 indicates greater than average death rate while numbers below 1 indicate a lower-than-average rate. For example, 1.2 indicates a 20 percent greater risk than average while 0.8 indicates a 20 percent lower risk.

### 2.2. Analyses of Phthalate

Nurses provided spot urine samples (2 × 2 mL) at the end of the work shift, (work duration at least 8 h per shift), and the day after the previous shift, which gives information about individual exposures during the last 24 h. We used high-performance liquid chromatography (HPLC) and tandem mass spectrometry (MS/MS) (Infinity 1260 and 6410 triplequad, Agilent, Santa Clara, CA, USA) to quantified urinary concentration of compounds: mono-methyl phthalate (MMP), mono-ethyl phthalate (MEP), mono-isobutyl phthalate (MiBP), mono-n-butyl phthalate (MnBP), mono(hydroxy-iso-butyl) phthalate (OH-MiBP), mono(hydroxy-n-butyl) phthalate (OH-MnBP), mono-benzyl phthalate (MBzP), mono-cyclohexyl phthalate (MCHP), mono-pentyl phthalate (MnPeP), mono-2-ethylhexyl phthalate (MEHP), mono(2-ethyl-5-hydroxyhexyl) phthalate (5OH-MEHP), mono(2-ethyl-5-oxohexyl) phthalate (5oxo-MEHP), mono(2-ethyl-5-carboxypentyl) phthalate (5cx-MEPP), mono-octyl phthalate (MnOP), mono-isononyl phthalate (MiNP), mono(oxo-methyl-octyl) phthalate (oxo-MiNP), mono(carboxy-methyl-heptyl) phthalate (cx-MiNP) by the method built on the basis of previously published off-line SPE and on-line HPLC-MS/MS methods [22,23]. The analysis was performed in Physiological Analytical Laboratory, Constantine the Philosopher University in Nitra, which has participated in the HBM4EU QA/QC programme, and its successful performance has resulted in its qualification as HBM4EU laboratory for the analysis of MEP, MBzP, MiBP, MnBP, MEHP, 5OH-MEHP, 5oxo-MEHP, 5cx-MEPP and cx-MiNP in human urine. Within this testing, we obtained very satisfactory Z-scores for previously meant compounds ranging from −1.1 to 0.7. The interlaboratory tests conditions for a successful passing were the Z scores ≤ |2|. Detailed description of these tests is summarized in a paper written by Esteban López et al. [24]. Internal quality control was performed by analyses of 2 control materials (mixture of urine samples) with known concentrations (lower and higher concentration). The limits of quantification (LOQ) were estimated between 1 and 2.5 ng/mL.

### 2.3. Statistics

For the description of levels of urinary phthalate metabolites, the means with standard deviations (SDs), medians, and the 5th and 95th percentiles of concentrations were computed. Associations between anthropometric parameters (BMI—Body Mass Index, WHR-Waist to Hip Ratio, WHtR-Waist to Height Ratio, FMI-Fat Mass Index, FFMI—Fat-Free Mass Index, ABSI—a Body Shape Index, HI—Hip Index, z BMI, z ABSI, z HI, BMI risk, ABSI risk, HI risk, ARI risk) and concentrations of phthalate metabolites were examined by Pearson correlation analysis. Pearson partial correlation was used to explain the association between phthalate metabolites and anthropometric parameters adjusted to consumer behavior (physical activity; consuming meals heated in plastic containers in the microwave, consuming meals from the plastic container, drinking beverages from plastic cups or bottles, using personal protective equipment, presence of polyvinyl chloride flooring material)and consumer practices (using: hand cream, antiperspirant, perfume, body lotion, nail polish; consuming: margarine, cheese, and salaams packaged in plastic material, meat products, baguette, salads, biscuits, and chocolate) during last 24 h before sampling. Multivariate regression analysis was used to estimate the association between anthropometric parameters and occupational exposure to phthalates, monitored by urinary phthalate concentrations log-transformed to base 10. All statistical analyses were performed using the SPSS for Windows statistical package (version 14.0; SPSS Inc., Chicago, IL, USA). A difference was considered statistically significant when *p* ≤ 0.05.

## 3. Results

The study population consisted of females (*n* = 50; 100%) with a mean age of 48.12 ± 10.74 years. Descriptive statistic of the study population is shown in Table 1.

Analysis of urine samples detected the presence of phthalate metabolites above LOQ of MEP in each urine sample. The presence of metabolites above LOQ in urine samples exceed the 90% limit in the case of MnBP, cx-MEPP (96%), of MiBP, of OH-MEHP (94%), of OH MnBP (92%), and oxo-MEHP a cx-MINP (90%), followed by the presence of phthalate metabolites OH-MiBP in 70% of samples, of MEHP and MBzP in 40% of samples, of oxo-MiNP in 38% of samples; of MnOP in 6% of samples and MCHP and MiNP in 2% of samples. The concentration of MnPeP was below the limit of quantification in all samples (100%) from the examined cohort. Descriptive statistics of urinary phthalate metabolites are shown in Table 2.

We observed associations between mono-benzyl phthalate (MBzP) and BMI risk (r = 0.49, *p* ≤ 0.001),MBzP, mono-isobutyl phthalate (MiBP), mono-hydroxy-iso-buthyl phthalate (OH-MiBP), mono-n-butyl phthalate (MnBP), mono-hydroxy-n-buthyl phthalate (OH-MnBP), mono-2-ethylhexyl phthalate (MEHP) and Hip index risk (HI risk; r = 0.3–0.44; *p* ≤ 0.04 respectively). Phthalate metabolites (MBzP, MiBP, MnBP, OH-MnBP), was associated with Anthropometric Risk Index (ARI risk; r = 0.32–0.33; *p* ≤ 0.03). MBzP was also associated with BMI, HC, WC, WHtR, Fat mas index (FMI), FFMI and visceral fat content (r = 0.31–0.55; *p* ≤ 0.033). Association was detected between mono-carboxy-isononyl phthalate (cx-MiNP) and Body mass index, hip circumference (HC), waist circumference (WC), Waist to height ratio (WHtR), and Fat free mass index (FFMI) (r = 0.31–0.4; *p* ≤ 0.03).

Using stepwise multiple linear regression analysis, we evaluated how the phthalate metabolites could affect the anthropometric parameters. Table 3 shows a significant relationship between BMI and levels of MBzP (β = 0.655; *p* < 0.001) and MEHP (β = −0.365; *p* = 0.003). We observed a similar pattern in the case of zBMI and BMI-risk. Furthermore, we found significant relationship between hip circumference and levels of MBzP (β = 0.486; *p* < 0.001), MEHP (β = −0.402; *p* = 0.001) and ∑DiNP secondary metabolites (β = 0.307; *p* = 0.016). We observed a similar pattern in the case of waist circumference, WHtR, FMI, and FFMI (Table 3). We found significant relationship between fat content and levels of MBzP (β = 0.302; *p* = 0.033), OH-MnBP (β = −0.736; *p* = 0.006) and MiBP (β = 0.547; *p* = 0.046); visceral fat content and levels of MBzP (β = 0.307; *p* = 0.030); HI-risk and levels of MBzP (β = 0.444; *p* = 0.001); ARI-risk and ∑DnBP (β = 0.337; *p* = 0.018).

We observed in addition to these results a statistically significant difference between phthalate concentrations in individuals who during the last 24 h consumed versus those who did not consume margarine (OH-MiBP 3.872 ng/mL vs. 1.479 ng/mL; r = 0.35, *p* = 0.012), sliced packaged cheeses (OH-MiBP 3.559 ng/mL vs. 1.433 ng/mL; r = 0.35, *p* = 0.013), packaged meat products (MEP 47.954 ng/mL vs. 22.140 ng/mL; r = 0.29, *p* = 0.042), and salami (MBzP 0.5 ng/mL vs. 0.78 ng/mL, r = 0.28, *p* = 0.052). Interestingly, we also observed a statistically significant higher concentration of cx-MiNP, depending on the use/non-use of protective equipment (vinyl medical gloves) (4.192 ng/mL vs. 2.319 ng/mL, r = −0.32, *p* = 0.025). On the other hand, we did not observe a statistically significant difference between concentration of phthalates and other aspects which monitored their consumer behavior (physical activity, heating and consuming meals from plastic container in microwave, drinking liquids from plastic caps or bottles, presence of polyvinyl chloride flooring material). In models adjusted to consumer behavior and consumer practices, we observed statistical significant associations between concentration of MBzP and BMI, HC, WC, WHtR, and FMI, visceral fat content, BMI risk and HI risk, adjusted to consumer behaviour and consumer practices (*p* ≤ 0.046, Table S1),

In same model, an association between metabolite MnBP and WHR (r = 0.36; *p* = 0.046), metabolite cx-MiNP and BMI (r = 0.37; *p* = 0.043), HC (r = 0.4; *p* = 0.026), WC (r = 0.35; *p* = 0.051) and WHtR (r = 0.38; *p* = 0.037), between oxo -MiNP and HC (r = 0.36; *p* = 0.045), between MEHP and ABSI (r = 0.36; *p* = 0.05), HI risk (r = 0.41; *p* = 0.022), and between oxo-MEHP and HI risk and ARI risk (r = 0.38–0.4; *p* ≤ 0.04) were detected.

## 4. Discussion

The main objective of our study was not only biomonitoring of occupational phthalate exposure of nurses but the assessment of obesity-related anthropometric indices of the participants based on actual measurements (not self-reported).

Results from biomonitoring of occupational phthalates exposure studies from Slovakia and worldwide [25] indicates lower exposure in current study. As expected, we observed temporal trends-declines in concentrations metabolites of phthalates that have been the focus of legislative activities in past. On the other hand, after comparison with our study from 2020 [26], nowadays we can observe an increase in the median MiNP, which is used as a DEHP substituent. These results correspond well with other findings that observed changes in phthalate exposure in the last decade in the general population. Although exposures to DnBP, BBzP, and DEHP have declined, exposures to replacement phthalates such as DiNP and DiBP have increased [27].

The main challenge when protecting high-risk groups in acute respiratory syndrome coronavirus 2 (SARS-CoV-2) situation is to increase the use of protective equipment. On the one hand, what appears positive can also have negative consequences, which applies to medical devices manufactured with the addition of plasticizers. Biocidal active substances, except others, pose as potential occupational health hazards. In a study analyzing hospital indoor air, PAEs (phthalate esters) pollution was ubiquitous. PAEs concentration in air varied widely between different hospital departments (hospitals’ drugstores, transfusion rooms, nurses’ workstations, wards, doctors’ offices, and halls), and the pollution of hospitals was more severe than that of newly decorated homes [28]. A study from 2021 also mentioned the migration of plasticizers (DEHP, DEHT, and DINP) from vinyl gloves is worrying due to direct dermal contact and can contribute to greater concern with possible toxic effects in the human body [29]. There also exists evidence of the occurrence of phthalates and non-phthalate plasticizers in facemasks [30].

The cx-MINP is secondary metabolite of DiNP, which belongs to the plasticizer used as a substituent of DEHP [31] in the manufacturing of many vinyl (polyvinyl chloride/PVC) products, including general-purpose and medical examination gloves [32].

The concentration of this metabolite in our study was associated with the use of protective equipment (vinyl medical gloves).

Association of the metabolite cx-MiNP, with BMI reported in our work agree with a former study [33]. Associations between cx-MINP and other anthropometric parameters (z BMI, hip circumference, waist circumference, WHTR, and FFMI) observed in our study, therefore, suggest hypothetical linking with the metabolism of fat tissue (which deserves more attention in further studies). The information mentioned above is supported by the observation in which exposure to DiNP and DiDP (di-iso-decyl-phthalate) in mice resulted in increased lipid accumulation in 3T3-L1 adipocytes, an effect likely mediated through activation of PPARγ and interference at different levels with the transcriptional cascade driving adipogenesis [34]. Additionally, in the context of the potential adipogenic activity of phthalates, we observed an association between MBzP and BMI, HC, WC, WHtR, FMI, visceral fat content, BMI risk, HI risk, and ARI risk. An association between MBzP and BMI is consistent with Amin et al. [35]. In our study, ARI risk and HI risk was also associated with concentration of MiBP, MnBP, and OH-MnBP. Moreover, benzyl butyl phthalate (BBP) promoted the differentiation of 3T3-L1 through the activation of pathways in adipogenesis and metabolic disturbance [36].

Our findings differ from the results of the National Nutrition and Health Survey (NHANES) from 1992–2002, as well as from the study by Hatch et al. [37], presenting mainly the metabolite MEP in a negative association with BMI. In contrast to our previous study [38], showing a statistically significant correlation between FFMI and MiNP, in the current study, we observed a positive correlation with concentration of OH-MnBP (r = 0.30), MBzP (r = 0.32), MMP (r = 0.3) and cx-MiNP (r = 0.32). When comparing with our former results [39], noting statistically significant associations between the metabolite MEHP with WHtR, WHR, waist circumference, and hips, we did not show any statistically significant relationships in this work. On the other hand, in the current study, we observed an association between oxo MEHP and HI risk.

Discrepancies mentioned above may have been affected by the specificity of the cohort, which, in addition to specific job classification, has consisted exclusively of women, who have significant differences in body parameters and consumer habits compared to men.

The etiology of obesity is multifactorial and depends on gene-environment interactions. EDCs may interfere with hormonal receptors that regulate adipogenesis and metabolic pathways [40]. Phthalates are associated with metabolic syndrome [41,42], to be linked with insulin resistance and type 2 diabetes [43], and obesity [35,44,45,46,47]. Observed were direct associations between phthalates (MiBP and MBzP) and adiposity-related traits, BMI, and waist circumference [48]. Animal and human fat cell models point to activation of PPAR-γ by MEHP, which results in stimulating adipogenesis [49,50]. In vitro evidence of MEHP effects on lipolysis, glucose uptake/glycolysis, and mitochondrial respiration/biogenesis has been published [51]. In conclusion, these findings support the theory that MEHP accumulation disturbs the energy metabolism of fat cells.

The results of stepwise multiple linear regression analysis might also point to the potential obesogenic effect of phthalates via their potential receptor-mediated activity. However, it is still unclear if they affect adipose tissue metabolism, and further study is needed to confirm or refute this hypothesis.

In evaluating the individual health risk, the case of BMI risk, HI risk, and ARI risk are important, namely where exposure to phthalates is associated with a risk of complications that affect quality and length of life (such as metabolic syndrome, diabetes mellitus, etc.). We hypothesize that not only exposure to specific phthalates and their concentration, but also the ratio between the concentration of specific phthalates would act in potential obesogenic effect as well as in other metabolic, reproductive, and neurobehavioral contexts.

We hypothesize that, although there were lower concentrations of phthalates in our study (in comparison to concentrations worldwide), which are commonly used in the manufacture of disinfectants, antimicrobials, cleaning products, biocides, personal care products, and biomedical materials (i.e., the daily occupational environment of nurses), long-term exposure scenarios could result in the obese-related findings observed in our study. This is only a hypothesis since the limitation of our study is also the absence of diester analyses from the air and medical devices. An aspect that we should take into account is also the higher age of the nurses in our study. Most were at an age when the perimenopausal transition occurs [52]. This suggests a hormonally sensitive period associated with changes in body weight. In a study from 2021, authors observed associations between MEHP, MEHHP, and MEOHP and one-year BMI change in women who transitioned from peri-to post-menopause from baseline to first follow-up [53]. In another study, phthalate metabolites were positively associated with hormones (estradiol, progesterone, and FSH) in premenopausal women [54]. The results of our study therefore hypothetically support the assumption that phthalates can act obesogenically (i.e., behave differently depending on their concentration and period of life when individuals are more hormonally sensitive).

Since some EDCs may interfere with hormonal receptors that regulate adipogenesis and metabolic pathways, we hypothesize that several phthalate esters act as agonists and/or antagonists via estrogen, androgen receptors, and PPARs [40,55,56]. The ratio between individual phthalates can be crucial for understanding different effects at the molecular level, and further studies are needed to elucidate this theory. Obviously, the synergic effect with other substances with endocrine-disrupting character and different routes of exposure has to be taken into account (lifestyle, consumer practices, etc.).

The other relevant aspect being discussed in the context of phthalates exposure is consumer behavior and consumer practices. One of these is practices related to meat packaging. In our study, individuals who consumed packaged meat products during the last 24 h compared to those who did not consume them had statistically higher concentrations of MEP (meat) and MBzP (salami, in which supports this hypothesis, in agreement to detection of high concentrations of phthalates in non-frozen packaged meat products [57].

In this study, we also observed increased concentrations of OH-MiBP in individuals who consumed sliced packaged cheeses during the last 24 h, and increased OH-MiBP in those who consumed margarine, compared to slightly higher concentrations of BBzP (8.4/g/mL) and DMP (11.7 µg/mL) compared to those consuming dairy products (eggs, milk, cheese) [58].

The results mentioned in the previous paragraphs deserve the attention of further studies in the context of associations between the concentration of MBzP and BMI, HC, WC, WHtR, and FMI, visceral fat content, BMI risk, and HI risk, an association between metabolite MnBP and WHR, cx-MiNP, and BMI, HC, WC, and WHtR, also between oxo -MiNP and HC, between MEHP and ABSI, HI risk, and between oxo-MEHP and HI risk and ARI risk, adjusted to the consumer behavior and consumer practices *p* ≤ 0.046) observed in our study.

Further, the ability of DBP and MEHP to penetrate mineral waters was detected, showing that the intake of fluids packaged in plastic containers increases exposure to phthalates and endangers the health of the exposed person [59] in conformity to the presence of DBP, DMP, DEP, and BBP in mineral bottled waters stored at room temperature [60]. Similar results were achieved by monitoring the presence of migration of DBP, DMP, DEP, and DnOP from plastic bottles to bottled water [61]. This is in contrast to our findings, in which we did not observe associations between the concentration of phthalates and consumer behavior (e.g., physical activity, heating and consuming meals from the plastic container in the microwave, drinking liquids from plastic caps or bottles, presence of polyvinyl chloride flooring material).

We hypothesize the results of our study support the hypothesis that the prevalence of obesity, besides being well-known, may be supported by several many lesser-known environmental factors that act in parallel [62].

Our study has several limitations. The participants were recruited from one region of Slovakia. Hence, the results should be applied cautiously to other populations. On the other hand, in terms of cohort uniformity, it is an advantage. Limited understanding of confounding factors, making up lifestyle, and modifying the concentration-effect associations, has to be taken into account. There also exists evidence that exposure to artificial lighting can directly affect circadian rhythms, resulting in weight gain and obesity not only in animals [63] but in humans, as well [64,65,66,67]. Nighttime illumination and disruption of circadian rhythms due to night shifts may be one of the factors influencing the development of obesity in our cohort. Further studies are needed to determine whether the results are consistent under different criteria.

## 5. Conclusions

The results suggest a hypothetical non-monotonic dose-response obesogenic activity of phthalates, demonstrated by the increase of abdominal obesity among nurses and estimated through novel anthropometric indices (such as ABSI, HI, or ARI), pointing to a real increase of health risk in perimenopausal transition. The present findings also emphasize the need for monitoring chemicals in the workplace of nurses as synergic effects with other chemicals and stressors could result in obesity-related diseases. The small sample size was one of the limitations of our study to realize these results. For this reason, our results cannot be generalized to the broader community based on this study alone.

Risk reduction of phthalate occupational exposure may be attained by the use of non-toxic alternatives. In this context, healthcare facilities and professionals present an important step in substituting of hazardous chemicals due to an ethical responsibility to use less hazardous products for patients and less purchasing power.

As an alternative to this measure, the development and implementation of a health-related physical fitness intervention program can promote and improve health and promote the working efficiency of nurses. The prevention, based on wellness and physical exercise paid by an employer as compensation for occupational health risk, can be efficient.

## Figures and Tables

**Table 1:** Descriptive characteristic of the cohort.

Anthropometric Parameter					Percentile		
	Mean	Median	MIN	MAX	5	95	SD
**Body height (cm)**	164.79	163.43	155.06	197.93	157.33	172.33	6.99
**Body weight (kg)**	71.45	68.15	55.10	122.50	57.20	107.60	14.61
**BMI (kg/m^2^)**	26.32	25.14	20.64	48.44	20.86	37.16	5.26
**Hip circumference (cm)**	103.63	102.50	90.00	148.33	92.00	128.33	10.55
**Waist circumference (cm)**	93.32	91.66	58.00	133.00	77.00	120.66	13.52
**WHR**	0.89	0.90	0.54	1.16	0.81	0.98	0.08
**WHtR**	0.56	0.55	0.35	0.84	0.46	0.72	0.08
**FMI**	9.06	7.82	1.14	26.35	2.62	17.54	4.52
**Lean mass**	46.76	46.30	38.08	60.15	38.17	59.50	6.37
**FFMI**	17.26	16.55	11.29	22.09	14.59	21.66	2.41
**Visceral fat (%)**	6.46	6.00	1.00	16.00	1.00	12.00	3.39
**ABSI**	0.08	0.08	0.05	0.10	0.08	0.09	0.01
**HI**	104.95	105.05	91.46	114.05	97.90	112.68	4.25
**z BMI**	−0.18	−0.36	−1.05	3.05	−1.01	1.38	0.79
**z ABSI**	0.63	0.65	−5.54	3.36	−0.78	2.25	1.29
**z HI**	0.07	0.13	−3.11	2.01	−1.56	1.71	0.96
**BMI risk**	0.94	0.89	0.84	1.85	0.84	1.17	0.16
**ABSI risk**	1.14	1.08	0.86	1.65	0.87	1.65	0.23
**HI risk**	1.00	0.96	0.95	1.36	0.95	1.14	0.08
**ARI risk**	1.07	0.97	0.73	2.18	0.80	1.62	0.30

Note: BMI, body mass index; WHR, waist to hip ratio; WHtR, waist to height ratio; FMI, fat mass index; FFMI, fat-free mass index; ABSI, A body shape index; HI, hip index; z BMI, BMI adjusted for age and sex; z ABSI, ABSI adjusted for age and sex; z HI, HI adjusted for age and sex; BMI risk, the risk of premature mortality based on the body mass index; ABSI risk, the risk of premature mortality based on the A body shape index; HI risk, the risk of premature mortality based on the hip index; ARI risk, the risk of premature mortality based on the anthropometric risk index.

**Table 2:** Descriptive statistics of urinary phthalate metabolites (ng/mL).

Phthalate Metabolite						Percentile	
	Mean	Median	SD	MIN	MAX	5	95
**MMP**	27.54	0.50	183.21	0.50	1297.04	0.50	8.95
**MEP**	102.00	33.77	177.44	1.81	823.21	2.91	487.29
**MBzP**	1.16	0.50	1.55	0.50	10.60	0.50	3.16
**MiBP**	23.35	15.82	24.97	1.77	122.90	1.77	86.67
**OH-MiBP**	4.77	2.00	9.39	0.50	60.33	0.50	11.76
**MnBP**	39.62	25.54	46.68	1.77	272.11	5.40	112.78
**OH-MnBP**	9.25	5.91	10.46	0.70	48.61	0.70	36.34
**MEHP**	1.99	1.00	1.55	1.00	7.19	1.00	5.17
**OH MEHP**	14.62	6.70	40.85	0.70	291.93	0.70	33.37
**oxo MEHP**	5.66	4.13	5.72	0.70	34.88	0.70	14.49
**cx MEPP**	10.66	9.17	9.73	0.70	66.66	2.56	19.97
**MiNP**	0.79	0.75	0.26	0.75	2.60	0.75	0.75
**oxo MiNP**	1.59	0.75	1.58	0.75	6.89	0.75	6.71
**cx MiNP**	3.89	2.97	3.38	0.70	19.75	0.70	9.69
**MnPeP**	1.25	1.25	0.00	1.25	1.25	1.25	1.25
**MCHP**	1.19	0.50	4.90	0.50	7.24	0.50	0.50
**MnOP**	0.83	0.75	0.33	0.75	2.42	0.75	1.74

Note: MMP, mono-methyl phthalate; MEP, mono-ethyl phthalate; MBzP, mono-benzyl phthalate, MiBP, mono-isobutyl phthalate; OH-MiBP, mono-hydroxy-iso-buthyl phthalate; MnBP, mono-n-butyl phthalate; OH-MnBP, mono-hydroxy-n-buthyl phthalate; MEHP, mono-2-ethylhexyl phthalate; OH MEHP, mono(2-ethyl-5-hydroxyhexyl) phthalate; oxo-MEHP, mono(2-ethyl-5-oxohexyl) phthalate; cx-MEPP, mono(2-ethyl-5-carboxypentyl) phthalate; MiNP, mono-isononyl phthalate; oxo MiNP, mono-oxo-isononyl phthalate; cx MiNP, mono-carboxy-isononyl phthalate; mnPeP, Mono-n-pentyl phthalate; MCHP, mono-cyclohexyl phthalate; MnOP, mono-n-octyl phthalate.

**Table 3:** Stepwise multiple linear regression analysis of phthalate metabolites effect on the anthropometric parameters.

Anthropometric Parameter	Phthalate Metabolite	β (95% CI)	*p*
**BMI**	MBzP	0.655 (7.219; 15.064)	<0.001
	MEHP	−0.365 (−11.313; −2.558)	0.003
**Hip circumference (cm)**	MBzP	0.486 (8.123; 25.029)	<0.001
	MEHP	−0.402 (−24.170; −6.462)	0.001
	DiNP	0.307 (2.101; 19.085)	0.016
**Waist circumference cm)**	MBzP	0.497 (10.187; 33.283)	<0.001
	MEHP	−0.291 (−27.084; −1.310)	0.032
**WHtR**	MBzP	0.520 (0.068; 0.205)	<0.001
	MEHP	−0.312 (−0.168; −0.015)	0.019
**Fat content (kg)**	MBzP	0.302 (0.487; 20.047)	0.040
	OH-MnBP	−0.736 (−28.338; −5.101)	0.006
	MiBP	0.547 (0.279; 26.777)	0.046
**FMI**	MBzP	0.565 (4.592; 11.916)	0.000
	MEHP	−0.330 (−9.469; −1.295)	0.011
**FFMI**	MBzP	0.307 (0.321; 4.458)	0.025
	MMP	0.280 (0.055; 2.094)	0.039
**Visceral fat content**	MBzP	0.307 (0.338; 6.400)	0.030
**zBMI**	MBzP	0.600 (0.916; 2.138)	<0.001
	MEHP	−0.390 (−1.790; −0.426)	0.002
**BMI-risk**	MBzP	0.551 (0.150; 0.408)	<0.001
	MEHP	−0.309 (−0.318; −0.031)	0.019
**HI-risk**	MBzP	0.444 (0.047; 0.178)	0.001
**ARI-risk**	DnBP	0.337 (0.042; 0.429)	0.018

Note: BMI, body mass index; WHtR, waist to height ratio; FMI, fat mass index; FFMI, fat free mass index; z BMI, BMI adjusted for age and sex; BMI risk, the risk of premature mortality based on the body mass index; HI risk, the risk of premature mortality based on the hip index; ARI risk, the risk of premature mortality based on the anthropometric risk index; MMP, Mono-methyl phthalate; MiBP, mono-isobutyl phthalate; MBzP, Mono-benzyl phthalate; MEHP, Mono-2-ethylhexyl phthalate; OH-MnBP, Mono-hydroxy-n-butyl phthalate; DiNP, Sum of Mono-isononyl phthalate (MiNP), Mono-oxo-isononyl phthalate (oxo MiNP) and Mono-carboxy-isononyl phthalate (cx MiNP); DnBP, sum of Mono-n-butyl phthalate (MnBP), Mono-hydroxy-n-butyl phthalate (OH-MnBP).

## Data Availability

Data available on request due to restrictions eg privacy or ethical.

## Supplementary Materials

The following supporting information can be downloaded at: https://www.mdpi.com/article/10.3390/toxics10030143/s1, Table S1: Associations between phthalate metabolites on the anthropometric parameters adjusted to consumer behavior and consumer practices.
