# Supplementary material for: Risk of Abdominal Obesity Associated with Phthalate Exposure of Nurses
Source: Toxics. 2022 Mar 18;10(3):143. doi: 10.3390/toxics10030143 (PMC8951402; doi:10.3390/toxics10030143)
Supplement: Supplementary file 1 [file toxics-10-00143-s001.zip › toxics-1648658-supplementary.pdf]

toxics

MDPI

Supplementary Materials: Risk of Abdominal Obesity Associated with Phthalate Exposure of Nurses

Branislav Kolena, Henrieta Hlisníková, Ľubica Kečkéšová, Miroslava Šidlovská, Tomáš Trnovec, And Ida Petrovičová

Table S1. Associations between phthalate metabolites on the anthropometric parameters adjusted to consumer behavior and consumer practices.

metabolite		Anthropometric parameter BMI	HC	WC	WHR	WHtR	FMI	FFMI	VF	ABSI	HI	z BMI	z ABSI	z HI	BMI risk	ABSI risk	HI risk	ARI risk
MMP	r	-,041	-,041	-,101	-,107	-,020	-,214	,294	-,163	,002	,146	-,074	-,154	,166	,032	-,174	-,121	-,101
	p	,825	,825	,590	,565	,915	,249	,108	,382	,991	,434	,691	,407	,374	,865	,349	,516	,590
MEP	r	,123	-,005	-,032	-,046	-,003	,122	,021	-,124	-,236	-,189	,149	-,147	-,171	,181	-,050	-,039	,070
	p	,509	,978	,866	,805	,986	,514	,913	,508	,201	,310	,423	,429	,357	,329	,791	,837	,709
MBzP	r	,568	,547	,588	,254	,611	,450	,288	,439	,079	,127	,472	,008	,144	,361	-,061	,385	,211
	p	,001	,001	,000	,169	,000	,011	,117	,013	,672	,497	,007	,965	,438	,046	,746	,032	,255
MiBP	r	,071	,115	,228	,243	,215	-,010	,151	,103	,302	,096	-,010	,183	,122	,046	,163	,314	,209
	p	,703	,538	,217	,188	,246	,959	,416	,582	,099	,607	,958	,325	,513	,806	,382	,086	,259
OH-MiBP	r	,062	,073	,194	,244	,148	-,070	,239	,024	,211	-,040	-,010	,202	-,034	-,014	,156	,246	,115
	p	,741	,696	,296	,186	,428	,708	,196	,899	,254	,830	,959	,276	,856	,939	,402	,183	,539
MnBP	r	,083	,064	,253	,361	,240	,023	,117	,160	,328	-,042	-,001	,198	-,013	,032	,175	,303	,207
	p	,658	,733	,169	,046	,193	,903	,531	,391	,071	,822	,995	,285	,946	,863	,345	,098	,264
OH-MnBP	r	-,070	-,003	,103	,199	,058	-,239	,285	-,138	,299	,059	-,130	,280	,064	-,059	,256	,276	,229
	p	,710	,986	,580	,284	,758	,196	,120	,458	,102	,752	,487	,127	,731	,754	,165	,133	,216
MEHP	r	-,038	,006	,174	,294	,145	-,018	-,040	,126	,355	,042	-,110	,244	,070	-,115	,200	,410	,157
	p	,840	,974	,350	,108	,436	,924	,831	,499	,050	,821	,555	,186	,710	,537	,280	,022	,399
OH MEHP	r	,058	,137	,136	,051	,147	-,017	,139	,045	,157	,200	-,027	,051	,248	-,046	,038	,084	-,001
	p	,758	,463	,464	,785	,430	,928	,457	,811	,400	,281	,887	,786	,179	,806	,839	,653	,996
oxo MEHP	r	,088	,175	,264	,213	,239	,016	,138	,046	,338	,204	,021	,347	,209	,140	,297	,377	,395
	p	,636	,347	,151	,251	,194	,930	,458	,805	,063	,271	,911	,056	,260	,454	,104	,036	,028
cx- MEPP	r	,134	,153	,178	,098	,217	,130	,028	,181	,147	,164	,038	,042	,189	,131	,021	,220	,150
	p	,472	,412	,337	,601	,240	,487	,883	,329	,432	,378	,838	,821	,308	,483	,911	,235	,420
oxo MiNP	r	,257	,363	,264	-,072	,222	,085	,336	,169	-,054	,204	,247	-,081	,230	,175	-,137	-,102	-,102
	p	,164	,045	,151	,701	,230	,648	,065	,362	,773	,271	,180	,666	,213	,347	,461	,585	,584
cx-MiNP	r	,366	,400	,354	,040	,376	,216	,315	,194	-,004	,205	,309	-,039	,219	,266	-,089	,140	,103
	p	,043	,026	,051	,830	,037	,244	,084	,295	,984	,268	,091	,836	,236	,148	,634	,451	,580

Note: MMP, mono-methyl phthalate; MEP, mono-ethyl phthalate; MBzP, mono-benzyl phthalate, MiBP, mono-isobutyl phthalate; OH-MiBP, mono-hydroxy-iso-buthyl phthalate; MnBP, mono-n-butyl phthalate; OH-MnBP, mono-hydroxy-n-buthyl phthalate; MEHP, mono-2-ethylhexyl phthalate; OH MEHP, mono(2-ethyl-5-hydroxyhexyl) phthalate; oxo-MEHP, mono(2-ethyl-5-oxohexyl) phthalate; cx- MEPP, mono(2-ethyl-5-carboxypentyl) phthalate; oxo MiNP, mono-oxo-isononyl phthalate; cx MiNP, mono-carboxy-isononyl phthalate; BMI, Body Mass Index; HC, hip circumference; WC, waist circumference; WHR, Waist to Hip Ratio; WHtR, Waist to Height Ratio; FMI, Fat Mass Index; FFMI, Fat-Free Mass Index; VF, visceral fat; ABSI, A Body Shape Index; HI, Hip Index; z BMI, BMI adjusted for age and sex; z ABSI, ABSI adjusted for age and sex; z HI, HI adjusted for age and sex; BMI risk, the risk of premature mortality based on the Body Mass Index; ABSI risk, the risk of premature mortality based on the A Body Shape Index; HI risk, the risk of premature mortality based on the Hip Index; ARI risk, the risk of premature mortality based on the Anthropometric Risk Index;.Adjusted to: physical activity; consuming meals heated in plastic containers in the microwave; consuming meals from the plastic container; drinking beverages from plastic cups or plastic bottles; using personal protective equipment (vinyl medical gloves); presence of polyvinyl chloride flooring material; using: hand cream; antiperspirant; perfume; salaams packaged in plastic material; meat products; baguette; salads; biscuits; chocolate during body lotion; nail polish; consuming: margarine; cheese and last 24 hours before sampling.
